# Supplementary material for: Reflective multi-immersion microscope objectives inspired by the Schmidt telescope
Source: Nat Biotechnol. 2023 Mar 30;42(1):65–71. doi: 10.1038/s41587-023-01717-8 (PMC10791577; doi:10.1038/s41587-023-01717-8)
Supplement: Supplementary file 2 — Reporting Summary [file 41587_2023_1717_MOESM2_ESM.pdf]

nature portfolio

Corresponding author(s): Fabian F. Voigt

Last updated by author(s): Feb 10, 2023

Reporting Summary

Nature Portfolio wishes to improve the reproducibility of the work that we publish. This form provides structure for consistency and transparency in reporting. For further information on Nature Portfolio policies, see our Editorial Policies and the Editorial Policy Checklist.

Statistics

For all statistical analyses, confirm that the following items are present in the figure legend, table legend, main text, or Methods section.

n/a	Confirmed	
☐	☒	The exact sample size (*n*) for each experimental group/condition, given as a discrete number and unit of measurement
☐	☒	A statement on whether measurements were taken from distinct samples or whether the same sample was measured repeatedly
☒	☐	The statistical test(s) used AND whether they are one- or two-sided<br>*Only common tests should be described solely by name; describe more complex techniques in the Methods section.*
☒	☐	A description of all covariates tested
☒	☐	A description of any assumptions or corrections, such as tests of normality and adjustment for multiple comparisons
☐	☒	A full description of the statistical parameters including central tendency (e.g. means) or other basic estimates (e.g. regression coefficient) AND variation (e.g. standard deviation) or associated estimates of uncertainty (e.g. confidence intervals)
☒	☐	For null hypothesis testing, the test statistic (e.g. *F*, *t*, *r*) with confidence intervals, effect sizes, degrees of freedom and *P* value noted<br>*Give P values as exact values whenever suitable.*
☒	☐	For Bayesian analysis, information on the choice of priors and Markov chain Monte Carlo settings
☒	☐	For hierarchical and complex designs, identification of the appropriate level for tests and full reporting of outcomes
☒	☐	Estimates of effect sizes (e.g. Cohen's *d*, Pearson's *r*), indicating how they were calculated

Our web collection on statistics for biologists contains articles on many of the points above.

Software and code

Policy information about availability of computer code

Data collection	ScanImage 2017b12 (Vidrio Technologies) running on MATLAB 326 2019b (MathWorks) was used to control multi-photon image acquisition with the Schmidt objective. mesoSPIM datasets were acquired using mesospim-control 1.7.2 (https://github.com/mesoSPIM/mesoSPIM-control), a custom open-source image acquisition software.
Data analysis	Data preprocessing (conversion to .tif, cropping, downsampling etc) was done using Fiji (fiji-2.0.0-pre-7). Volume renderings were generated with Imaris 9.6.0 (Bitplane) and exported supplemental videos were edited with Premiere Pro CC 2021 (Adobe). PSF datasets were analysed using Python 3.8.0 and napari 0.4.16. Calcium imaging datasets were analysed using suite2p v0.10.0 (https://github.com/MouseLand/suite2p).

For manuscripts utilizing custom algorithms or software that are central to the research but not yet described in published literature, software must be made available to editors and reviewers. We strongly encourage code deposition in a community repository (e.g. GitHub). See the Nature Portfolio guidelines for submitting code & software for further information.

Data

Policy information about availability of data

All manuscripts must include a data availability statement. This statement should provide the following information, where applicable:

- Accession codes, unique identifiers, or web links for publicly available datasets
- A description of any restrictions on data availability
- For clinical datasets or third party data, please ensure that the statement adheres to our policy

The data that support the findings of this study are available from the corresponding author upon request.

Human research participants

Policy information about studies involving human research participants and Sex and Gender in Research.

Reporting on sex and gender	The sample was obtained from a female patient.
Population characteristics	The patient (Age: 82 years) was diagnosed with Alzheimer's disease, dementia, aphasia, and depression.n/a
Recruitment	The human occipital lobe samples were obtained from the body donation program of the Department of Anatomy and Embryology, Maastricht University. The tissue donor gave their informed and written consent to the donation of their body for teaching and research purposes as regulated by the Dutch law for the use of human remains for scientific research and education ("Wet op de Lijkbezorging"). Accordingly, a handwritten and signed codicil from the donor posed when still alive and well, is kept at the Department of Anatomy and Embryology Faculty of Health, Medicine and Life Sciences, Maastricht University, Maastricht, The Netherlands.
Ethics oversight	All methods were carried out in accordance with the relevant guidelines and regulations and all experimental protocols were approved by the Ethics Review Committee Psychology and Neuroscience (ERCPN).

Note that full information on the approval of the study protocol must also be provided in the manuscript.

Field-specific reporting

Please select the one below that is the best fit for your research. If you are not sure, read the appropriate sections before making your selection.

☒ Life sciences ☐ Behavioural & social sciences ☐ Ecological, evolutionary & environmental sciences

For a reference copy of the document with all sections, see nature.com/documents/nr-reporting-summary-flat.pdf

Life sciences study design

All studies must disclose on these points even when the disclosure is negative.

Sample size	We did not calculate sample sizes beforehand. Regarding resolution measurements: Microscope point spread-functions are highly reproducible as they are caused by deterministic physical processes such as wavefront propagation and aberrations. Therefore, typical publications estimate PSFs with small sample sizes. To estimate PSF parameters reliably over the full FOV (equivalent to zoom 1), we chose the bead concentration such that 8 beads could be measured across sub-FOVs acquired using zoom 40.
Data exclusions	Only PSFs from beads surrounded by a bead-free window of 2 x 2 x 5 microns (X x Y x Z) were included in the analysis.
Replication	Fitting a PSF (for FWHM estimation) to each individual bead represents an individual measurement.
Randomization	We chose random subregions of the test samples (cover slips coated with beads) for PSF measurements.
Blinding	As the person analyzing the bead data was the same who took the data, blinding was not possible.

Reporting for specific materials, systems and methods

We require information from authors about some types of materials, experimental systems and methods used in many studies. Here, indicate whether each material, system or method listed is relevant to your study. If you are not sure if a list item applies to your research, read the appropriate section before selecting a response.

Materials & experimental systems

n/a	Involved in the study
☐	☒ Antibodies
☒	☐ Eukaryotic cell lines
☒	☐ Palaeontology and archaeology
☐	☒ Animals and other organisms
☒	☐ Clinical data
☒	☐ Dual use research of concern

Methods

n/a	Involved in the study
☒	☐ ChIP-seq
☒	☐ Flow cytometry
☒	☐ MRI-based neuroimaging

Antibodies

Antibodies used	Mouse anti-neurofilament (RMO270, Invitrogen 13-0700)<br>Polyclonal rabbit-anti-tyrosine hydroxylase antibody (Sigma Aldrich, AB152)<br>Polyclonal chicken-anti-GFP antibody (Aves Labs, GFP-1020)<br>Donkey-anti-rabbit-AlexaFluor594 antibody (ThermoFisher, A32754)<br>Donkey-anti-chicken-AlexaFluor594 antibody (Jackson Immuno Research, 703-585-155)<br>Atp1a1 antibody (DSHB, A5)<br>Alexa Fluor-594 antibody (ThermoFisher A32742)<br>Goat anti-mouse IgG-Alexa 568 (Jackson ImmunoResearch 115-165-003)
Validation	Antibody validation information for the Invitrogen 13-0700 mouse anti-neurofilament antibody is available from Thermo-Fisher Scientific under the Catalog-Number #13-0700. Validation information for the Sigma-Aldrich AB152 13-0700 and DSHB Atp1a1 antibody is available from the Sigma-Aldrich and DSHB, respectively.

Animals and other research organisms

Policy information about studies involving animals; ARRIVE guidelines recommended for reporting animal research, and Sex and Gender in Research

Laboratory animals	elavl3:GCaMP6s zebrafish larvae, 5 days old.<br>C57BL6/J 363 wildtype and Thy1-YFP HJrs/J transgenic (Jackson, 003782) mice aged 4-17 weeks.<br>Xenopus tropicalis tadpoles,4 days old
Wild animals	The study did not involve any wild animals.
Reporting on sex	The study used female mice. Sex of zebrafish and xenopus larvae can not be determined during the early stages we imaged.
Field-collected samples	The study did not involve any field-collected samples.
Ethics oversight	Cantonal Veterinary Office of the Canton of Zurich

Note that full information on the approval of the study protocol must also be provided in the manuscript.
